# The Strengths of r- and K-Selection Shape Diversity-Disturbance Relationships

**DOI:** 10.1371/journal.pone.0095659

**Published:** 2014-04-24

**Authors:** Kristin Bohn, Ryan Pavlick, Björn Reu, Axel Kleidon

**Affiliations:** 1 Biospheric Theory and Modelling Group, Max Planck Institute for Biogeochemistry, Jena, Germany; 2 Ecological Modelling, Friedrich-Schiller-University, Jena, Germany; 3 Department of Special Botany and Functional Biodiversity Research, University Leipzig, Leipzig, Germany; University of Pennsylvania, United States of America

## Abstract

Disturbance is a key factor shaping species abundance and diversity in plant communities. Here, we use a mechanistic model of vegetation diversity to show that different strengths of r- and K-selection result in different disturbance-diversity relationships. R- and K-selection constrain the range of viable species through the colonization-competition tradeoff, with strong r-selection favoring colonizers and strong K-selection favoring competitors, but the level of disturbance also affects the success of species. This interplay among r- and K-selection and disturbance results in different shapes of disturbance-diversity relationships, with little variation of diversity with no r- and no K-selection, a decrease in diversity with r-selection with disturbance rate, an increase in diversity with K-selection, and a peak at intermediate values with strong r- and K-selection. We conclude that different disturbance-diversity relationships found in observations may reflect different intensities of r- and K-selection within communities, which should be inferable from broader observations of community composition and their ecophysiological trait ranges.

## Introduction

The level of disturbance is one of many factors that shape patterns of plant species diversity [1]. The Intermediate Disturbance Hypothesis (IDH) [2], [3] states that the diversity of a community is greatest at intermediate levels of disturbance. This peak in diversity is hypothesized to result from the contrasting effects of disturbance on the ability of species to persist in a community. Greater rates of disturbance require more rapid recolonization of sites, which should exclude species that are slow in reproduction, thus decreasing the diversity in a community. However, with decreasing rates of disturbance, strong competitors increasingly outcompete weaker competitors, resulting in increased levels of competitive exclusion and lower levels of diversity. Thus, the combination of the two effects suggest a peak in diversity at intermediate levels of disturbance, resulting in a unimodal disturbance-diversity relation (e.g. [4], [5]).

The IDH and, more generally, disturbance-diversity relationships (DDRs) can be interpreted as the outcome of how well species are able to compete and colonize under different rates of disturbances. This ability is not arbitrary, but constrained by the colonization-competition tradeoff to which all plant species in a community are subject to. Plant species differ in their traits, which influence their competitive and colonizing abilities. A plant species that produces high biomass has a greater ability to harvest light and reach other resources and thus has a strong competitive ability. Such a strategy is favored by K-selection [6] In contrast, a plant strategy that allocates more to reproduction instead of biomass can rapidly establish on free sites, thus having a greater colonizing ability. Such a strategy is favored by r-selection [6]. Since plants cannot be both strong competitors and quick colonizers, they are subjected to the fundamental tradeoff between allocation to biomass and reproduction. Hence, the colonization-competition tradeoff is seen as the main mechanism that results in the IDH (e.g. [7]). Although we constrain our discussion here to plants, it should in general apply to other organisms, e.g. animals, because the colonization-competition tradeoff should also apply to them.

The universality of the IDH has been tested for a wide range of empirical systems (e.g. [8]–[11]) and with theoretical studies (e.g. [4], [12]). The unimodal DDR associated with the IDH has not been consistently observed (summary in [10]). The relationship of diversity with disturbance rate can also be U-shaped, increasing or decreasing, or even insignificant (e.g. [10], [13]). Several alternative explanations have been proposed to explain these different shapes. Huston [14], Proulx and Mazumder [15] and Kadmon and Benjamini [16] explained different DDRs with an interdependence of productivity between different species. Following this idea, a unimodal relationship between diversity and disturbances can be realized only under intermediate levels of productivity (e.g. [16], [17]). They suggest that with high productivity, diversity increases with disturbances, while under low productivity, diversity decreases with disturbances. However, productivity and diversity are not independent variables, but reflect different aspects of the environment, the disturbance regimes as well as the level of competition within communities. Due to such inconsistencies, Fox [18] has suggested to abandon the IDH. However, in this paper we do not want to reject or support the IDH, rather we seek a general theory to explain the different shapes of DDRs, which is still missing [19].

Here, we hypothesize that different shapes of DDRs result from different strengths by which r- and K-selection exclude species from plant communities. We test this hypothesis with a model in which the strength of r- and K-selection can indirectly be adjusted, for instance by the disturbance rate. The composition of the modelled plant community, e.g. in terms of the relative abundances of colonizers and competitors, results from different independent processes: climatic constraints, disturbance, and competitive processes. We refer to strong K-selection if we observe a high presence of competitors. This is usually the case under high resource competition. We refer to strong r-selection if we observe high presence of colonizers, which is usually the case under strong seed competition. Seed and resource competition can be adjusted in the model independently varying two parameters. By altering the seed and resource competition in the model, the resulting community reflects different strength of r- and K-selection. We therefore refer to the strength of r- and K-selection and not to seed and resource competition.

**Figure 1:**
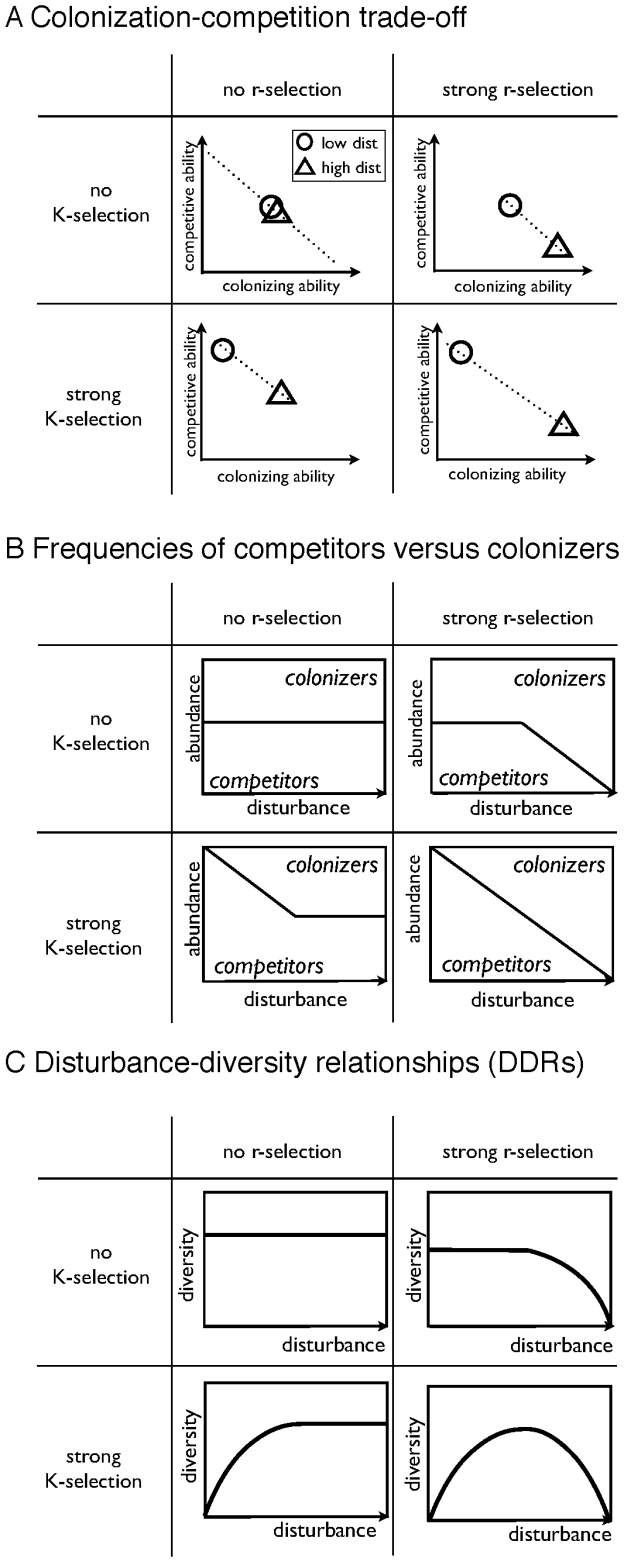
Different strength of r- and K-selection lead to exclusion of different species and thereby result in different DDRs. In the absence of r- and K-selection, no species are excluded, and the colonization-competition tradeoff is expected to have the widest range. K-selection shifts this tradeoff towards competitive species, while r-selection shifts this tradeoff towards colonizers. Under both r- and K-selection, the tradeoff (A) is hypothesized to have a wider range, because it shifts from competitors towards colonizers under increasing disturbances. The abundances of colonizers versus competitors (B) reflect these relationships and lead to different shapes of DDRs (C): flat, increasing, decreasing and unimodal.

With no competitive exclusion, the neutral theory of biodiversity [20] assumes that differences among species are irrelevant to their success, so that neither r- nor K- selection exclude species from a community. At this extreme, the competition-colonization tradeoff allows species over a broad range of traits to persist in the community (Fig. 1 A), because r- or K-selection do not exclude sections of this tradeoff. Since the success of species is independent of their differences, we hypothesize that colonizers and competitors should be present at a similar abundance (Fig. 1 B), thus resulting in a flat DDR (Fig. 1 C). We expect that K-selection results in a reduction of the range of the competition-colonization tradeoff due to competitive exclusion. Thus, we expect colonizers to be absent at low disturbances and become increasingly present at higher disturbance rates. We hypothesize that this isolated effect of K-selection results in an increase in diversity to a saturating value with increasing rates of disturbance. With r-selection, we expect to find the competition-colonization tradeoff to be reduced towards the other end, favoring colonizers at the expense of strong competitors. We propose that this results in a greater abundance of colonizers towards higher disturbance rates, but also reduces the diversity at this end because competitors are increasingly excluded. Thus we expect diversity to decline with greater disturbance rates. When both r- and K-selection are considered, we hypothesize to find a less reduced range of the competition-colonization tradeoff and a continuous shift in the abundance from competitors to colonizers, resulting in the unimodal DDR that is associated with the IDH.

To evaluate these hypotheses, we use a plant physiology-based numerical model of plant diversity (JeDi-DGVM [21], [22]) in combination with a model of population dynamics (DIVE [23]). The JeDi-DGVM tests a wide range of plant growth strategies for their reproductive success under different climatic conditions and thereby represents a mechanistic climate filter [24]. It has been used successfully in previous studies to understand biogeographical patterns of plant species richness [25], relative abundance distributions [26], as well as vegetation productivity [22]. The model is used here as a mechanistic way to obtain the range of the colonization-competition tradeoff for a given climatic setting. The DIVE model [23] represents a competitive filter, in which we can vary the strength of r- and K-selection as well as disturbance rate and evaluate their effects on the resulting simulated diversity of the community. With this we estimate the effects of r- and K-selection on the resulting DDRs.

In the following, we first provide a brief description of the two models in the methods section, describe the setup as well as the analysis of simulations for the sensitivity analysis. The simulation results are presented in a similar way as the line of reasoning was presented in Fig. 1. We then describe potential limitations of this approach and interpret the results in terms of our hypothesis and close with a brief summary and conclusion.

## Methods

**Figure 2:**
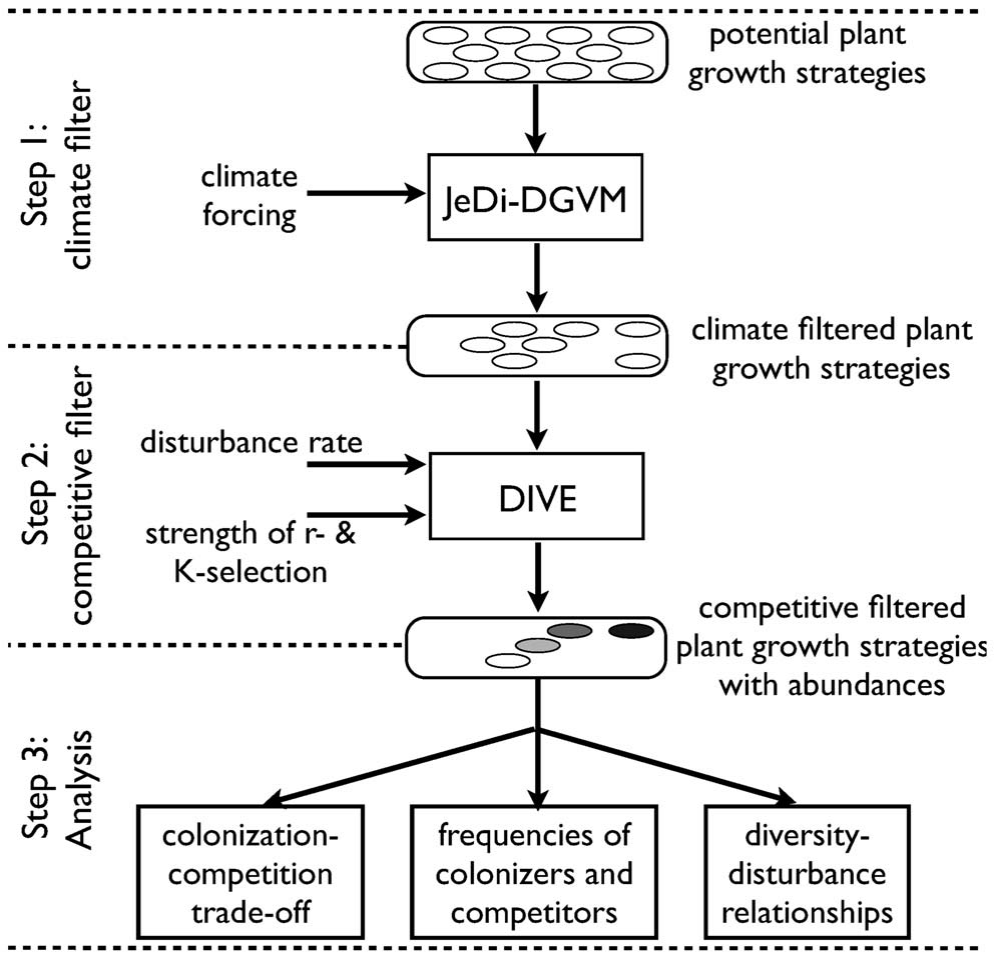
Schematic diagram of our approach, in which we use two simulation models. The first model, the JeDi-DGVM, represents the climatic filter that estimates the plant growth strategies that are potentially able to be reproductively successful in a given climatic environment. The second model, the DIVE model, simulates the population dynamics of these plant growth strategies and represents a competitive filter. The strength of r- and K-selection and the rate of disturbance are external model parameters. DIVE calculates the abundance of the plant growth strategies, from which the colonization-competition tradeoff, the abundance of colonizers and competitors and the diversity-disturbance relationships are being derived.

We use two simulation models to test our hypotheses. The first model tests many hypothetical plant growth strategies for their success under given climatic conditions, acting as a ‘climate filter’. Then, these successful growth strategies are applied to a second model, which acts as a ‘competitive filter’. In a third step this data is analyzed. The approach is summarized in Fig. 2.

### Step 1: The climate filter

**Figure 3:**
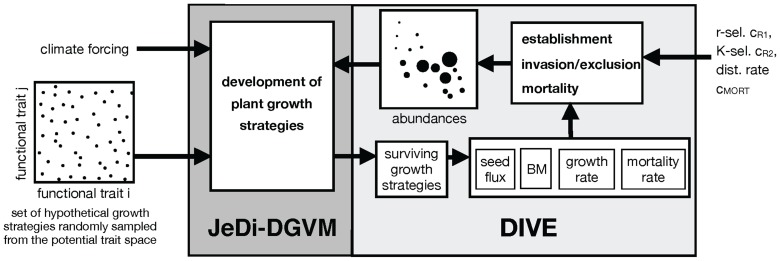
Schematic diagram of JeDi-DGVM (adopted from [22]) and DIVE. JeDi-DGVM simulates the development of many randomly sampled hypothetical plant growth strategies. Each plant growth strategy consists of trait parameters, that characterize plant carbon allocation, phenology, and other ecophysiological attributes. The plant growth strategies develop based on fundamental ecophysiological processes (e.g. photosynthesis and allocation) and the environmental conditions. The environmental conditions are provided by land surface processes, which are simulated out of the climatic conditions. Functional tradeoffs and the prevailing climatic conditions ultimately determine the relative performance of the growth strategies. DIVE uses the performance parameters of the surviving growth strategies to calculate rates of establishment, invasion and exclusion and mortality, which in turn results in the relative abundance of each growth strategy in the community. Thereby r-selection, K-selection and disturbances affect the spatial dynamics. The resulting abundances feed back into JeDi-DGVM.

The first step represents the climate filtering of a wide variety of potential plant growth strategies for their reproductive success. This filter is implemented by the Jena Diversity-Dynamic Global Vegetation Model (JeDi-DGVM, see Fig. 3 left, [21], [22]). JeDi-DGVM is developed out of the individual-based KM2000 model [21]. JeDi-DGVM does not model individuals, but rather populations of individuals. JeDi-DGVM simulates plants as plant growth strategies in terms of several carbon pools associated with leaves, stem, roots, and reproduction and in terms of their physiological processes of photosynthesis, respiration, resource allocation to different biomass pools as well as reproduction, and phenology. The simulated processes are affected by the climatic conditions and by a set of functional trait parameters. The climatic condition, particularly solar radiation, temperature and soil moisture are used to simulate land surface processes, such as canopy interception, infiltration, evaporation, root water uptake, and runoff. The land surface parameters (e.g. leaf area index, surface albedo, and rooting depth) are derived from the biomass pools and from the functional trait values of each growth strategy. The functional trait paramater values are randomly sampled from a potential trait space comprising the theoretical ranges of 15 functional trait parameters. These functional traits include, for instance, the relative allocation to different carbon pools, ecophysiological tradeoffs, and phenological responses and thereby control plant growth and life history. Competition between growth strategies, however, is not simulated by the model.

We simulated a large number of trait combinations of randomly chosen values using a realistic climatic forcing and evaluated these combinations for their reproductive success. These successful strategies were then used in Step 2.

### Step 2: The competition filter

The second step represents the explicit simulation of population dynamics using the successful plant growth strategies from Step 1. This step uses the DIVE model [23], which is a simple representation of population dynamics. It has been previously shown to adequately reproduce successional patterns [23].

DIVE is based on the concept of theoretical population dynamics models (see e.g. [27]), while processes are represented more mechanistically and the demographic parameters in more realistic (see [23] for full model description). The DIVE model simulates the abundances of the successful plant growth strategies. The abundance of a plant growth strategy is represented as differential equation (see Eq. 1) of the change in occupied area 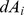 based on a species establishment 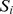 on bare ground, invasion 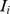 and exclusion 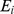 by competition for occupied area and mortality 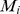.
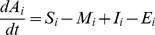
(1)Each of these processes itself is represented by a differential equation. Since the invasion and exclusion depend on the occupied area of the affected species, the differential equations cannot be solved analytically. Therefore DIVE has to be calculated step by step.

The simulated population dynamics (eq. 1) are affected by the performance (Step 1) of the different plant growth strategies. For instance, the simulated allocation to reproduction and the growth rate determine its colonizing ability - thereby growth rate is a surrogate on how fast a plant reaches adult size. Further, the biomass turnover relates to the mortality of a strategy, and the simulated biomass of a particular strategy determines its competitive ability. Hence competition for resources is modeled implicitly by biomass dominance and results in K-selection. As an approximation, we assume that size matters, in that larger plants will typically outcompete smaller ones, e.g. due to being better competitors for light, water or others resources (e.g. [28]). Of course, this assumption may not be true for all cases, but in general it should.

We used several performance traits [29], such as biomass, seed flux, productivity, and biomass turnover, to derive demographic parameters, such as growth and mortality rates for the population dynamics (see [23] for details).

In addition, the dynamics of establishment, competition, and mortality are affected by externally prescribed parameters relating to the strengths of r- and K-selection as well as the disturbance rate. The strength of r-selection is mediated by a parameter 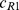, which affects the importance of the seed flux in the rate of establishment of a particular plant growth strategy. In the absence of r-selection (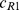 is high) the magnitude of the seed flux of a particular strategy does not influence the rate of its establishment. Under strong r-selection (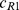 is 1), the strategy with the highest seed flux has the highest potential to establish. The realized establishment rate is also dependent on the growth rate, so that a colonizer, or r-strategist, is characterized by a high seed flux and a high growth rate. The strength of K-selection is mediated by a parameter 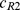, which determines the importance of the biomass of a strategy for competitive exclusion. In the absence of K-selection (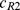 is high) differences in biomass among strategies do not result in competitive exclusion. With strong K-selection (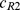 is 1), exclusion is proportional to the difference in biomass among different strategies (with the assumption that strategies with higher biomass are more dominant). Hence, a strong competitor, or K-strategist, is characterized by a high biomass. The disturbance rate is a parameter 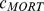 which acts to uniformly increase the mortality and thereby reduces the abundance of all simulated strategies. The higher the disturbance rate, the greater the reduction in abundance. The disturbance rate is modeled in this simple way, because we are interested in the general effects of disturbances. The continuous rate captures different disturbances in one rate, e.g. disease, herbivory, grazing, fire, windfall. This usage stays in contrast to the definition of White and Pickett [30], who defined disturbance as “any relatively discrete event”. However, looking at the big picture over larger areas, discrete disturbance events might considered a continuous phenomena. Further, modeling all disturbance types in a process-based way is still not feasible in models. The continuous disturbance rate used here allows us to investigate the general effects of disturbances.

### Step 3: Analysis

The simulated abundances from Step 2 are analyzed to infer the colonization-competition tradeoff, the relative abundance of competitors and colonizers, and the diversity of the simulated community to test the hypotheses shown in Fig. 1.

The colonization-competition tradeoff is derived directly from the different successful plant growth strategies which pass both the climate and competitive filters. We use the simulated biomass of these strategies as an indicator for the competitive ability of a strategy, and the simulated growth rate as an indicator for its colonizing ability.

**Figure 4:**
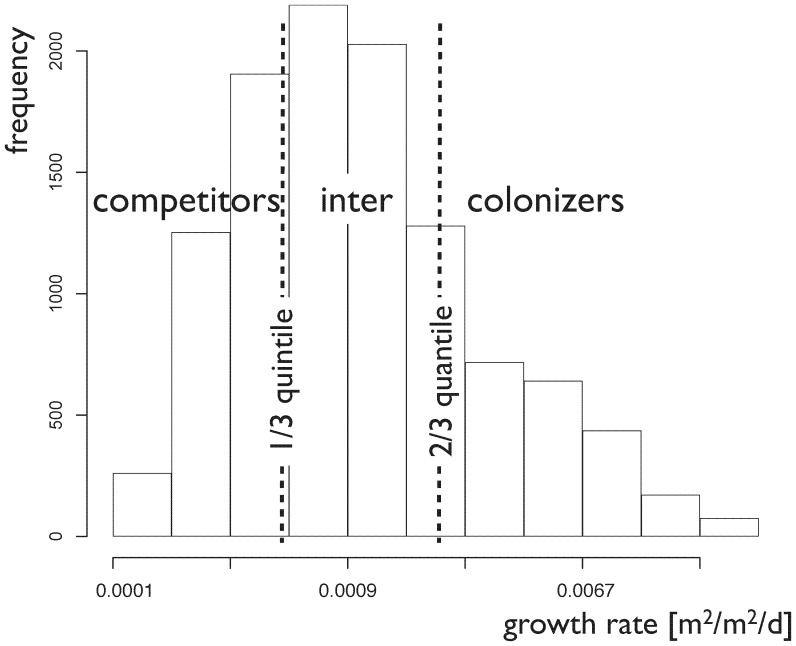
The successful plant growth strategies simulated by the model are characterizes as competitors, colonizers and intermediates dependent on their growth rate. We use three quantiles of the distribution of growth rates from all simulations, as shown by the dashed lines, for the partitioning.

To classify the simulated strategies in terms of competitors or colonizers, we use the distribution of simulated growth rates of the different strategies as a basis (see Fig. 4). We define colonizers to be strategies that are in the top 1/3 quantile of the distribution, while competitors are taken to be those strategies that are in the bottom 1/3 quantile. We refer to the middle quantile as intermediate strategies. We want to clarify at this point, that of course all strategies compete, but we use this classification to especially refer to strategies having a better colonizing or competitive ability.

To infer DDRs, we measured the diversity of the resulting steady state community. In our model setup, a steady state community is reached, while under disturbances this is not expected. However, under disturbances stable coexistence, which means that abundances do not show long-term trends [1], can occur, especially when applying on the large scale. From a stable coexisting community diversity can be calculated, in this way the reader should understand the steady state community. Diversity is calculated as Shannon information entropy (H) from the relative abundances of the successful strategies (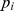): 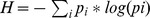. This measure has the advantage that species with a very low abundance contribute very little to diversity, in contrast to other metrics like species richness. We normalized the diversity by the maximum diversity across all the simulated communities, resulting in the so called evenness. The evenness allows to compare results easily, because the scales are set between zero and one.

### Simulation setup

We used a climatic forcing representative of a moist tropical climate with a mean daily precipitation of 9 mm/day and a mean temperature of 26.3°C. We tested a set of 500 initial randomly-sampled plant growth strategies. The JeDi-DGVM was run for 70 years, so that the characteristics of each strategy represent the mean properties of an adult population. The simulated properties were used to run the DIVE model. The simulated abundances from the DIVE model were returned to the JeDi-DGVM at a monthly time step to establish a feedback between population dynamics and the computation of seed production in the JeDi-DGVM (but see Fig. 3). This setup was run for a total of 100000 years to ensure steady state composition of the community.

We conducted a number of simulations for the sensitivity analyses with this model setup by modifying the parameters that mediate the strengths of r- and K-selection (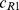 and 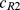) as well as the disturbance rate 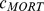. Three levels of r- and K- selection were used, representing none, moderate, and strong strength (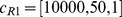 and 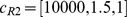 respectively, see [23] for details]). We used five disturbance rates from low to high (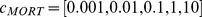 respectively). In total, this resulted in 45 model simulations.

## Results

In the presentation of the results, we follow the sequence shown in Fig. 1. We first show the simulated colonization-competition tradeoff for different strengths of r- and K-selection, the abundance of colonizers and competitors, and finally the simulated diversity-disturbance relationships (DDRs).

### The colonization-competition tradeoff

**Figure 5:**
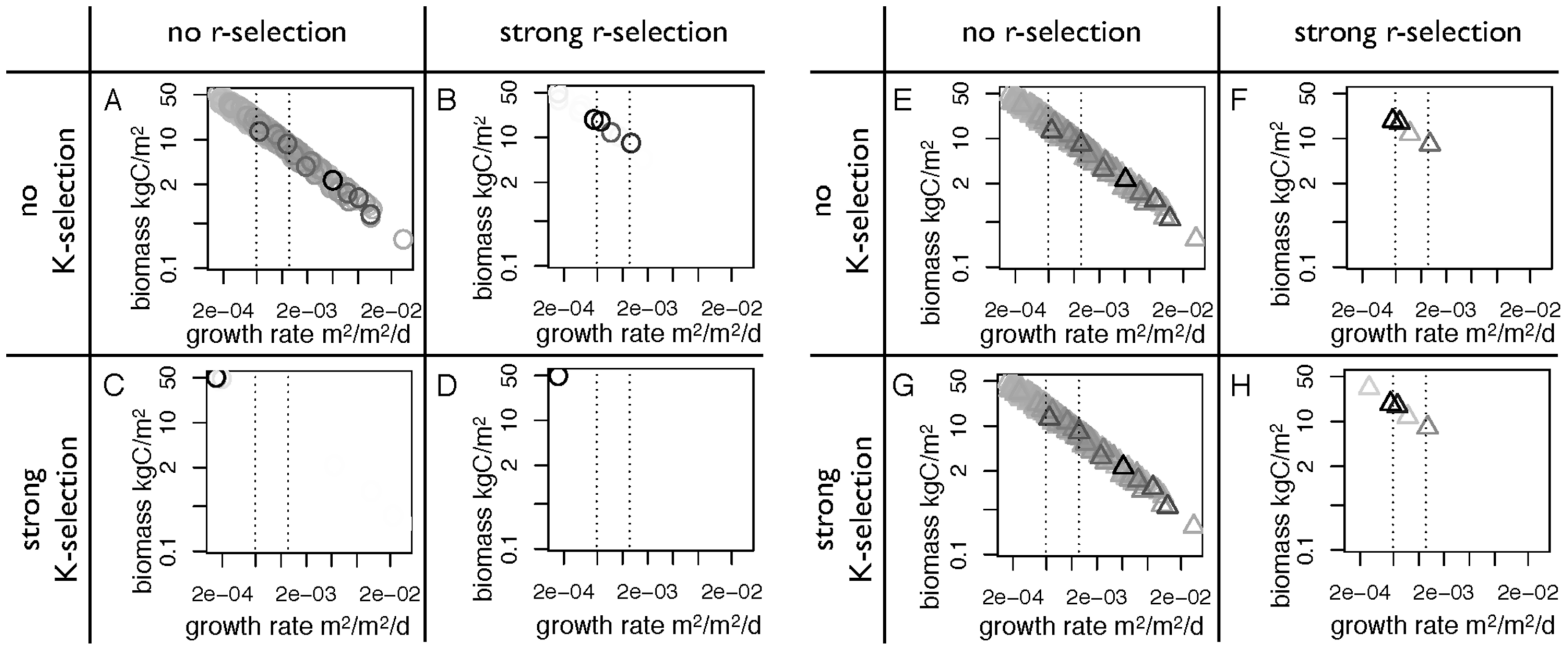
Sensitivity of the simulated colonization-competition tradeoff to r- and K-selection. Every symbol in the figure reflects a successful plant growth strategy. The grey scale indicates the normalized abundance of these strategies from low abundance (white) to high abundance (black). Circles represent the simulations of low disturbance rates (a–d), while triangles represent the simulations with high disturbance rates (e–h). The dotted lines show the breaks for colonizers, intermediates and competitors.

The simulated colonization-competition tradeoff for different strengths of r- and K-selection for low and high disturbance rates is shown in Fig. 5. In the absence of both r- and K-selection, the colonization-competition tradeoff spans the widest range (Fig. 5a and e). Since neither competitive nor colonizing ability can increase the abundance of a particular growth strategy due to the lack of r- and K-selection in the model, the simulated abundances are entirely determined by the growth and mortality of the different growth strategies. The colonization-competition tradeoff is very similar in terms of range and abundances under low and high disturbances. The most abundant strategy in both cases is classified as a colonizer.

When r-selection is included in the simulations, the tradeoff is strongly constrained to a narrow range (see Fig. 5b and f). Both strong competitors as well as quick colonizers are excluded in the simulations, and the resulting strategies are mostly classified as intermediates. In the model, this results from the combined need of high seed production as well as high growth rate to be an effective colonizer.

With strong K-selection, the range of the tradeoff is hardly different compared to the case of no r- and no K-selection (Fig. 5c and g). In contrast to the case of no r- and K-selection, the strategies of highest abundance is clearly different in the cases of low and high disturbance. In the case of low disturbance, the most abundant strategy is a strong competitor with high biomass and low growth rate. In the case of high disturbance, the most abundant strategy is a colonizer with a relatively high growth rate.

With both strong r- and K-selection, the range of the tradeoff is reduced compared to the case of no r- and K-selection (Fig. 5d and h), but wider compared to the case of only r-selection. The most abundant strategy in the case of low disturbance rate is a strong competitor as in the case of only K-selection, while in the case of high disturbance, the most abundant strategy is shifted along the tradeoff towards a stronger colonizing ability.

Overall, we find that the model simulates the colonization-competition tradeoff very well. The model results broadly support the expected differences in the tradeoff under different settings of r- and K-selection and disturbance. K-selection shifts the most abundant strategy towards a strong competitor with a low disturbance rate, and towards a better colonizer with a high disturbance rate. With respect to r-selection, we find a somewhat different behavior, because the tradeoff is shifted less towards colonizing ability as hypothesized. This can be attributed to the way that the model describes establishment. The highest rate of establishment is achieved in the model by strategies that have high seed production in addition to a relatively high growth rate, whereas the number of produced seeds is not explicitly treated in the model. Nevertheless, the range of the tradeoff is reduced with r-selection as hypothesized.

### Abundances of colonizers, intermediates, and competitors

**Figure 6:**
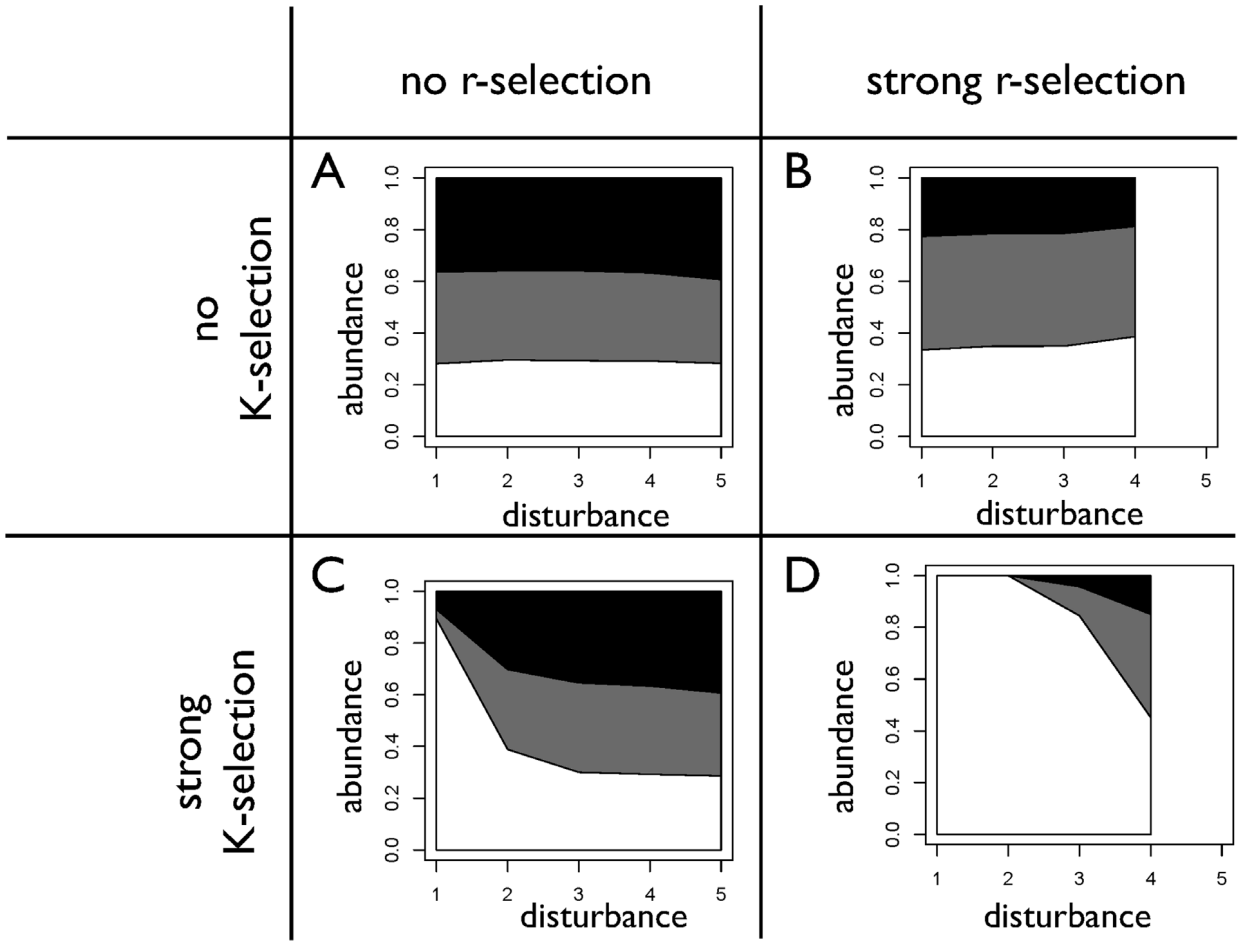
Abundances of competitors (white), intermediates (grey), and colonizers (black) for the different scenarios of r- and K-selection and disturbance rate. In the case of strong r-selection and high disturbance rate (level 5), no strategies survive so that no relative abundances are shown for this disturbance rate.

The differences in composition in terms of colonizers, intermediates, and competitors for the different scenarios of r- and K-selection and the sensitivity to disturbance rates are shown in Fig. 6. We find that with neither r- nor K-selection, neither colonizers nor competitors are favored under different disturbance rates, because of the lack of r- and K-selection in the model. This lack of sensitivity is consistent with the tradeoff being essentially identical under the different disturbance regimes shown in Fig. 5. With strong r-selection, the abundance of competitors is increased. This somewhat surprising result is nevertheless consistent with the reduction and shift of the tradeoff shown in Fig. 5. With strong K-selection, competitors have a higher abundance under low disturbance rates than the case of no r- and K-selection, and their abundance is reduced with increasing disturbance rates. Consequently, the abundance of colonizers is enhanced with higher disturbance rates. This is, again, consistent with the shift in abundance that was seen in the tradeoff in Fig. 5. When both r- and K-selection are included, competitors have the highest abundance at low disturbance rates, which successively decrease with greater disturbance rates. This sensitivity is again consistent with the tradeoff characteristics shown in Fig. 5.

In summary, we find that the simulated sensitivities of the abundances of competitors and colonizers are consistent with the hypothesized trends shown in Fig. 1 for the cases of no r- and K-selection and only K-selection. The results for r-selection deviate somewhat from the hypothesized trend, because the most favored strategies under r-selection are not the strategies with the highest growth rates. In the case of both r- and K-selection, we nevertheless find a trend that is somewhat consistent to our hypothesis.

### Diversity-disturbance relationships

**Figure 7:**
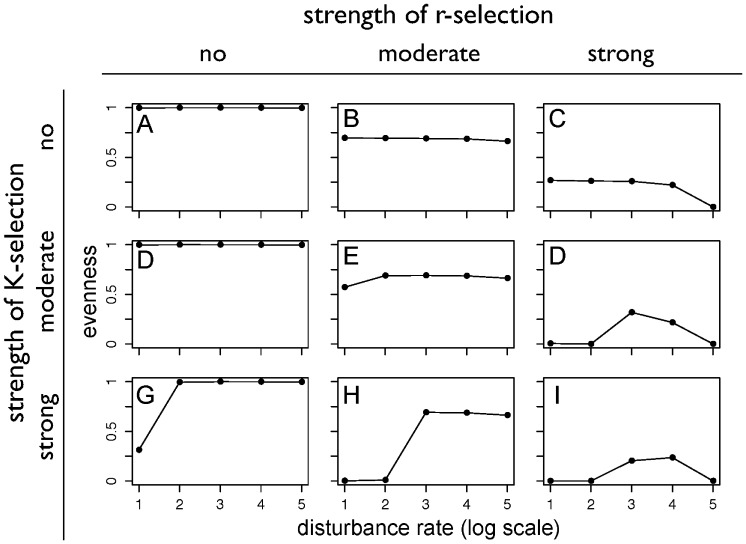
Simulated DDRs for different strengths of r- and K-selection.

The resulting shapes of the DDRs for the different scenarios are shown in Fig. 7. With no r- and K-selection, the simulated diversity of the community is insensitive to disturbance rate and remains at the maximum level of diversity. With increasing r-selection (Fig. 7a–c), we find a successive decrease in the diversity, and an increased sensitivity of diversity to high disturbance rates. In the case of strong r-selection, diversity is reduced at high disturbance rates, resulting in a decreasing DDR. This is consistent with the reduced range of the colonization-competition tradeoff shown in Fig. 5. With increasing strength of K-selection (Fig. 7a,d,g), we find that the diversity is first relatively unaffected, but then is reduced at low disturbance rates in the case of strong K-selection. Hence, this results in an increasing DDR at strong K-selection. The relative insensitivity of diversity to K-selection is consistent with the insensitivity of the range of the tradeoff axis shown in Fig. 5, while the reduction of diversity at low disturbance rates is consistent with the reduction of the tradeoff axis at strong K-selection at low disturbance rates. When both r- and K-selection, are considered, we note an increasing combination of the two effects. The maximum diversity reached in the different scenarios of r- and K-selection strength successively is reduced with increasing r-selection as is the diversity at high disturbance rates. Hence, the combination of r- and K-selection results in an unimodal DDR. This relationship is consistent with the differences in the tradeoff shown in Fig. 5. The range of the tradeoff with strong r- and K-selection is reduced at low disturbance rates, and is broadened in the case of high disturbance rates.

In total, we find that different shapes of the DDRs can be reproduced with different strengths of r- and K-selection. These different shapes can clearly be attributed to the effects that r- and K-selection have on the range of the colonization-competition tradeoff as represented in the model. This, in turn, is consistent with the hypotheses that we formulated in the introduction.

## Discussion

### Limitations

We used two different models, JeDi and DIVE, in our study. Each model by itself obviously has limitations that affect the results to some extent. A general discussion of the limitations of the models can be found in their respective publications [21]–[23]. Here, we focus on those aspects that directly relate to the testing of our hypotheses regarding the different shapes of the diversity-disturbance relationships (DDRs).

The JeDi-DGVM is the basis for our study, as it yields the potential range of the colonization-competition tradeoff that is compatible with the prescribed climatic conditions. The tradeoff is simulated via describing the competitive ability by the biomass, and the colonizing ability by the growth rate, which is proportional to biomass, and does not include seed production. This tradeoff is therefore simulated to be a relatively narrow line (see Fig. 5), which means that the assimilated carbon is either used for growth or colonization. However, real plants also use carbon for other processes than just growth, for instance defense and nutrient acquisition. These processes represent carbon allocation to different uses than the tradeoff, so that this could result in a tradeoff that is more spread out than what is being simulated by the JeDi-DGVM. Given that the JeDi-DGVM can reproduce a range of observations very well [21], [22], [25], [26], it is reasonable to assume that this tradeoff, despite probably being too constrained to a line, is nevertheless reasonably simulated.

This tradeoff represents the key input for the DIVE model, which, based on the representation of r- and K-selection, simulates the actual abundances of competitors and colonizers. The DIVE model was shown to reasonably represent successional dynamics in ecosystems [23], so that in principle, the effect of r- and K-selection should be adequately represented. We notice, however, that the abundances of competitors and colonizers with increasing disturbance rates (Fig. 6) did not fully follow the trends that we expected (as shown in Fig. 1). We attribute this to the way that colonization is represented in the DIVE model as a combination of seed production and growth rate. In this representation, the tradeoff between seed size and seed numbers is not considered. However, this tradeoff was found to be important [31], because it partly modulates the colonization-competition tradeoff and leads to species coexistence (e.g. [32], [33]). In our model, plants with a higher biomass may have a higher seed production than plants with a smaller biomass, so that the plants with the highest seed production may not be the ones with the smallest biomass. An explicit representation of this tradeoff could therefore result in a sensitivity to the strength of r-selection that would be more consistent with our hypotheses.

To obtain DDRs, a range of prescribed disturbance rates were simulated. Disturbances are modeled in a relatively simple way by a single parameter that influences the mortality equally across all plant growth strategies [23]. In the real world, disturbances represent a range of singular events, such as droughts, fire, herbivory, and wind throw. Furthermore, several disturbance types interact (summary in [34]), as well as the intensity and the frequency of disturbances [35], [36]. Despite the simplicity of our representation, the simulated sensitivities to disturbance rates are nevertheless plausible and consistent with our hypotheses, indicating that our representation captures the overall role of disturbances on the simulated abundances.

### Interpretation

Our results mostly support our hypothesis that different strengths of r- and K-selection shape different types of DDRs. We confirm our hypothesis that a flat relationship is obtained in the absence of r- and K-selection, because the abundances are then shaped entirely by the growth and mortality of the different plant strategies. Diversity decreases with disturbance with strong r-selection in our results mostly due to a decrease in overall diversity of the community. With strong K-selection, colonizers are less excluded at stronger disturbance rates, thus resulting in an increase of diversity with disturbance. With strong r- and K-selection, both effects are combined and yield an unimodal DDR.

Our interpretation of the role of r- and K-selection for DDRs is a straightforward and simple extension of the original work on the IDH [2], [3], in which the different strengths by which r- and K-selection act to exclude species from the composition of a community is being varied. By doing so, different strengths of the mechanism that results in the IDH is implemented, yielding different shapes of the DDR.

However, we do not explicitly state the mechanism by which such a difference in r- and K-selection could take place. One plausible explanation may be the spatial scale that is being considered. Tilman [27] showed that greater coexistence and diversity in communities is possible with the explicit consideration of space. Consistent with this interpretation, the neutral model of Hubbell [20] shows that increasing space leads to greater diversity. Combined, it would appear that a greater consideration of space makes the overall composition more neutral, corresponding to a lower strength of r- and K-selection acting on the composition. In addition, climate may also alter the strengths of r- and K-selection as well. These aspects would need to be further evaluated in future work.

Theoretical ecology offers a variety of hypotheses regarding the maintenance of biological diversity. Chesson [1] argues that to achieve coexistence equalizing and stabilizing mechanism are needed. Stabilizing mechanisms are essential for species coexistence, equalizing mechanism are also needed, but alone not sufficient. Stabilizing mechanism tend to increase negative intraspecific interactions relative to negative interspecific interactions, such as mortality. Equalizing mechanism minimize fitness differences between species.

In our study under specific conditions, stable coexistence is achieved. The existing environmental variations present stabilizing mechanism, because a variable environment prevents having only one best adapted plant strategy. Disturbances in our model can be adjusted (via 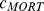), additional stabilizing mechanisms are therefore increasingly present in simulation with a higher disturbance rate. Equalizing mechanisms in our model are present through the colonization-competition tradeoff. If a species has a low competitive ability, then this is compensated by a higher colonizing ability. Following Chesson, this tradeoff alone is not able to lead to stable coexistence. This is confirmed in our simulations. In simulations under strong strength of r- and K-selection combined with low disturbances coexistence is not achieved. The high strength of selection makes the advantages of competitors and colonizers very important for their success. The species are not equal and stabilizing mechanism are not present to obtain coexistence, when disturbances are low. Increasing disturbance increases the stabilizing mechanism, leading to coexistence. Decreasing strength of r- or K-selection makes species more equal, and can also lead to coexistence. In the case of no r- and no K-selection, the advantages of competitors and colonizers decrease to zero. This means, that we can interpret the strength of r- and K-selection as controlling the effectiveness of the equalizing mechanism.

Our results are largely consistent with Johst and Huth [12]. They used a patch model of successional dynamics and found a unimodal DDR for most forest ecosystems under discrete disturbances. The unimodal DDR was generated through the successional order from early towards late successional species, where at intermediate disturbances a mixture of all successional stage species coexisted. Their results correspond to our scenario where r- and K-selection are present. Under such conditions we also observe an unimodal DDR but considering continuous disturbances. Under some circumstances Johst and Huth [12] also found a bimodal DDR. In their study, the occurrence of a species rich intermediate successional group led to a local minimum between the maxima of mixtures of successional groups. We did not find such a bimodal relationship, likely because we do not consider the diversity of successional groups and do not represent discrete disturbance events.

Our work is only partially consistent with the results of dos Santos et al. [37]. They used a spatially explicit individual-based model and showed that tradeoff mechanisms usually led to unimodal DDRs, while neutrality led to decreasing DDRs. Tradeoff mechanisms support the transition from pioneers towards late successional species, while neutral communities do not support this transition. The tradeoff mechanism supports succession only under r- and K-selection, which is consistent with our results. The neutral community in our study would be reflected in the absence of r- and K-selection, which led to a flat DDR. However, dos Santos et al. [37] also found a flat DDR for a neutral community of long dispersers with a negative density dependent recruitment. This negative density dependent mechanism of long dispersers corresponds to the absence of r-selection, thus being consistent with our results.

The study of Seifan et al. [34] provided an alternative mechanism for unimodal DDRs compared to ours. They used a demographic temperate grassland model and found an unimodal DDR but this was not generated by the colonization-competition tradeoff. In their study, tradeoffs between species-specific responses to disturbances [34] maintained diversity. However, in grasslands most species are colonizers, so that the whole range of the colonization-competition tradeoff was likely not considered in their study.

### Conclusion

We presented a hypothesis that explains different shapes of diversity-disturbance relationships (DDRs) by different strengths of competition. The colonization-competition tradeoff plays a key role in this hypothesis. A plant needs to trade, whether it invests into its competitive ability and grows tall but slowly (competitor) or whether it can establish rapidly but grows only small (colonizer). While r-selection favors colonizers, K-selection favors competitors. This results in four types of DDRs. A flat DDR is achieved in the absence of r- and K-selection. Diversity increases with disturbance under K-selection, and decreases under r-selection. An unimodal DDR is achieved under both r- and K-selection. We successfully tested our predictions with a process-based simulation model of tropical plant communities. Our results are consistent with other modeling studies on the effects of disturbances for diversity.

Our results show that different intensities of r- and K-selection have different effects on the range of the colonization-competition tradeoff, therefore affecting the abundances of colonizers versus competitors and thus influence community structure and the shape of the DDR. The strength of r- and K-selection in a community can thus be expected to be reflected in the combined information of abundances of colonizers versus competitors, the range of the colonization-competition tradeoff, the diversity, and possibly the spatial scale of observation as this may affect the strength of r- and K-selection. What this implies is that a broader range of field observations may help us to better identify the underlying mechanisms that result in observed diversity patterns.
